# Anti-Cancer Effect of Thiacremonone through Down Regulation of Peroxiredoxin 6

**DOI:** 10.1371/journal.pone.0091508

**Published:** 2014-03-11

**Authors:** Miran Jo, Hyung-Mun Yun, Kyung-Ran Park, Mi Hee Park, Dong Hun Lee, Seung Hee Cho, Hwan-Soo Yoo, Yong-Moon Lee, Heon Sang Jeong, Youngsoo Kim, Jae Kyung Jung, Bang Yeon Hwang, Mi Kyeong Lee, Nam Doo Kim, Sang Bae Han, Jin Tae Hong

**Affiliations:** 1 College of Pharmacy and Medical Research Center, Chungbuk National University, Chungbuk, Korea; 2 College of Agriculture, Life and Environments Science, Chungbuk National University, Chungbuk, Korea; 3 New Drug Development Center, Daegu-Gyeongbuk Medical Innovation Foundation, Daegu, Korea; University of Hawaii Cancer Center, United States of America

## Abstract

Thiacremonone (2, 4-dihydroxy-2, 5-dimethyl-thiophene-3-one) is an antioxidant substance as a novel sulfur compound generated from High-Temperature-High-Pressure-treated garlic. Peroxiredoxin 6 (PRDX6) is a member of peroxidases, and has glutathione peroxidase and calcium-independent phospholipase A2 (iPLA2) activities. Several studies have demonstrated that PRDX6 stimulates lung cancer cell growth via an increase of glutathione peroxidase activity. A docking model study and pull down assay showed that thiacremonone completely fits on the active site (cys-47) of glutathione peroxidase of PRDX6 and interacts with PRDX6. Thus, we investigated whether thiacremonone inhibits cell growth by blocking glutathione peroxidase of PRDX6 in the human lung cancer cells, A549 and NCI-H460. Thiacremonone (0–50 μg/ml) inhibited lung cancer cell growth in a concentration dependent manner through induction of apoptotic cell death accompanied by induction of cleaved caspase-3, -8, -9, Bax, p21 and p53, but decrease of xIAP, cIAP and Bcl2 expression. Thiacremonone further inhibited glutathione peroxidase activity in lung cancer cells. However, the cell growth inhibitory effect of thiacremonone was not observed in the lung cancer cells transfected with mutant PRDX6 (C47S) and in the presence of dithiothreitol and glutathione. In an allograft in vivo model, thiacremonone (30 mg/kg) also inhibited tumor growth accompanied with the reduction of PRDX6 expression and glutathione peroxidase activity, but increased expression of cleaved caspase-3, -8, -9, Bax, p21 and p53. These data indicate that thiacremonone inhibits tumor growth via inhibition of glutathione peroxidase activity of PRDX6 through interaction. These data suggest that thiacremonone may have potentially beneficial effects in lung cancer.

## Introduction

Peroxiredoxins (PRDXs) are a family of peroxidases as antioxidant enzymes [1]–[2]. The PRDX family includes six members. They are divided into two classes [3]. The 2-Cys group includes PRDX1-5, whereas PRDX6 is only a member of the 1-Cys group. PRDXs are a family of peroxidases that destroy peroxides using conserved cysteine residues in the catalytic center [4]. Among the six mammalian members of this family, PRDX6 is the only member that has glutathione peroxidase and calcium-independent phospholipase A2 (iPLA2) activities [5]. Whereas other PRDXs utilize thioredoxin as a physiological reductant, PRDX6 utilizes glutathione [6]. PRDX6 protects cells from membrane, DNA, protein damages, and lipid peroxidation [7]. The antioxidant response element (ARE) in the prdx6 promoter region, a *cis*-acting regulator element, is activated by oxidative stress [8]. Transcription of the PRDX6 gene is regulated by nuclear factor erythroid 2-related factors 1, 2, and 3 (Nrf1, Nrf2, and Nrf3) as transcription factors via binding to the ARE [9]. Among the Nrfs, Nrf2 positively regulates transcription of the PRDX6 gene [10].

As PRDXs are antioxidants, they support survival and tumor maintenance by protecting cells from oxidative stress-induced apoptosis [11]. In a recent study, over expression of PRDX 6 attenuates cisplatin-induced apoptosis in human ovarian cancer cells [12]. In contrast, reduction of PRDX6 expression increased peroxide-induced cell death in liver cancer cells [13]. The invasion and metastasis promoting actions of PRDX6 has been found in lung cancer cells through activation of Akt via activation of phosphoinositiede 3-kinase (PI3K) and p38 kinase [4], [14]. The activity of PRDX6 contributes to the metastatic ability of lung cancer cells by stimulating invasion components including PI3K, Akt, and uPA [4]. It was also reported that PRDX6 expression in lung cancer cells was significantly associated with tumor progression [15].

Garlic has been used in traditional medicine as a food component to prevent the development of cancer [16]. Thiacremonone (2,4-dihydroxy-2,5-dimethyl-thiophene-3-one) is an antioxidant substance, as a novel sulfur compound, generated from High-Temperature-High-Pressure (HTHP)-treated garlic [17]. In the present study, we investigated the anti-cancer effect of thiacremonone through the inhibition of glutathione peroxidase activity via interaction in lung cancer cells.

## Materials and Methods

### Extraction and characterization of thiacremonone

**Figure 1:**
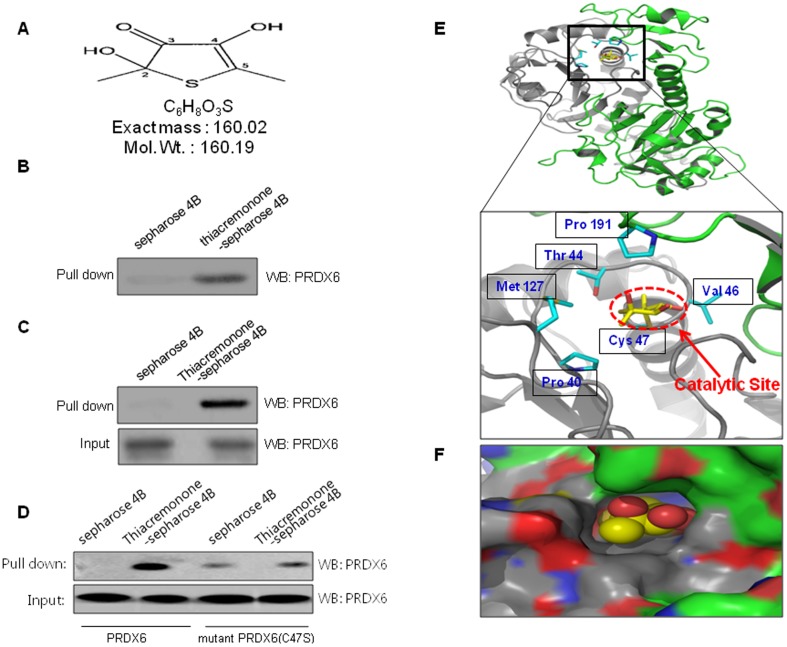
Structure of thiacremonone and PRDX6 and binding of thiacremonone to PRDX6. (A) Structure of thiacremonone, a sulfurcompound isolated from garlic. (B) Recombinant protein of PRDX6 was incubated with thiacremonone-conjugated Sepharose 4B. (C) Whole cell lysates of NCI-H460 were incubated with thiacremonone-conjugated Sepharose 4B. After precipitation, the levels of bound PRDX6 were monitored by Western blot analysis. (D) After precipitation with thiacremonone-conjugated Sepharose 4B, the levels of bound PRDX6 or mutant PRDX6 C47S were monitored by Western blot analysis. (E) Ribbon representation docking model of thiacremonone with PRDX6 (F) Molecular surface representation docking model of thiacremonone with PRDX6.

The structure of a sulfur compound isolated from garlic (named thiacremonone) is shown in results (Fig. 1A). Garlic (Allium sativum L) was heated at temperatures of 130°C for 2 hrs. The heated samples were juiced and then filtered on a Buchner funnel under a vacuum. Heated garlic juice was partitioned consecutively in a separating funnel using ethyl acetate. Isolation of the compounds from the ethyl acetate layer of heated garlic juice was subjected to column chromatography on silica gel. This fraction containing thiacremonone was purified by preparative RP-HPLC on a Younglin SP930D Instrument [17]. Thiacremonone was resolved in 0.01% dimethyl sulfoxide, and treated at concentrations of 10, 20, and 50 μg/ml in culture cells.

### Cell Culture

The A549 and NCI-H460 lung cancer cell lines were purchased from the American Type Culture Collection (Manassas, VA). The CCD-18Co colon normal cell line and LL24 lung normal cell line were purchased from the Korean cell line bank (Seoul, Korea). NCI-H460 and LL24 normal cells were cultured in RPMI-1640 medium supplemented with 10% fetal bovine serum (FBS) and penicillin/streptomycin (100 U/ml). A549 and CCD-18Co normal cells were cultured in a DMEM medium supplemented with 10% fetal bovine serum (FBS) and penicillin/streptomycin (100 U/ml). Cell cultures were then maintained at 37°C in a humidified atmosphere of 5% CO2.

### Cell Viability Assay

To determine the cell numbers, A549 and NCI-H460 lung cancer cells or CCD-18Co colon normal cell line and LL24 lung normal cell line were plated in 24-well plates (5×10^4^ cells/well). Subconfluent cells were subsequently treated with thiacremonone (10, 20, and 50 μg/ml) for 72 hr. After treatment, cells were trypsinized and pelleted by centrifugation for 5 min at 1,500 rpm, resuspended in 5 ml of phosphate-buffered saline (PBS), and 0.1 ml of 0.2% trypan blue was added to the cancer cell suspension in each of the solutions (0.9 ml each). Subsequently, a drop of suspension was placed into a Neubauer chamber and the living cancer cells were counted. Cells that showed signs of staining were considered to be dead, whereas those that excluded trypan blue were considered viable. Each assay was carried out in triplicate.

### Transfection

Lung cancer cells (5×10^4^ cells per well) were plated in 24-well plates and transiently transfected with PRDX6 siRNA (Santa Cruz Biotechnology) or pcDNA-PRDX6, (generous gifts from DR. Jhang Ho Pak, University of Ulsan College of Medicine using a mixture of Prdx6 siRNA or pcDNA-Prdx6 and the WelFect-EX PLUS reagent in OPTI-MEN, according to the manufacturer's specifications (WelGENE, Seoul, Korea).

### Luciferase Activity Assay

A549 and NCI-H460 human lung cancer cells were transfected with prdx6 promoter-Luc plasmid using a mixture of plasmid and the WelFect EX PLUS reagent in OPTI-MEN, according to the manufacturer's specifications (WelGENE, Seoul, Korea). After 6 hr, the cells were treated with thiacremonone. Luciferase activity was measured by using the luciferase assay kit (Promega, Wisconsin, USA) according to the manufacturer's instructions (WinGlow, Bad Wildbad, Germany).

### Western Blot Analysis

The membrane was incubated for 2 hr at room temperature with specific antibodies: rabbit polyclonal for PRDX6 and cIAP2 (1∶1,000 dilution, Abcam, plc. Cambridge UK), xIAP, Bcl2, caspase-3, caspase-9 (1∶1,000 dilution, Cell Signaling Technology, Inc., Beverly, MA), Bax (1∶500 dilution, Santa Cruz Biotechnology, Inc.) and mouse monoclonal for p53, and caspase-8 (1∶1,000 dilution, Cell Signaling Technology, Inc.), p21 (1∶500 dilution, Santa Cruz Biotechnology, Inc.). The blot was then incubated with the corresponding conjugated anti-rabbit and anti-mouse immunoglobulin G-horseradish peroxidase (1∶2,000 dilution, Santa Cruz Biotechnology, Santa Cruz, CA). Immunoreactive proteins were detected with ECL Western blotting detection system.

### Glutathione Peroxidase Activity Assay

GPx co-substrate mixture, including nicotinamide adenine dinucleotide phosphate (NADPH), glutathione, and glutathione reductase, was used to measure glutathione peroxidase activity in vitro. A GPx Assay Kit was purchased from Cayman Chemical (Michigan, USA) and procedures were done according to the manufacturer's instructions. After treating the cells, they were homogenized and subjected to the assay, and the absorbance value was measured at 340 nm and normalized to protein concentration.

### Molecular Modeling

The crystal structure of PRDX6 (PDB code 1prx) was used for the docking study. The thiacremonone was built using a Maestro build panel. The compound was minimized using the Impact module of Maestro in the Schrödinger Suite Program. The starting coordinate of the PRDX6 was further modified for binding model prediction. The protein structure was minimized using the Protein Preparation Wizard by applying an OPLS force field. For the grid generation, the binding site was defined as the centroid of the Cys 47 in the catalytic site of PRDX6. Ligand docking into the catalytic site of PRDX6 was carried out using the Schrödinger docking program, Glide. The minimized conformation of thiacremonone was docked into the prepared receptor grid. The best-docked poses were selected as the initial covalent model of Cys 47 in the catalytic site of PRDX6 with thiacremonone. The covalent complexes were further minimized using the steepest descent algorithm. Molecular graphics for the covalent binding model of the thiacremonone was generated using a PyMol package (http://www.pymol.org).

### Pull Down Assay

Thiacremonone was conjugated with cyanogen bromide (CNBr)-activated Sepharose 4B (Sigma-Aldrich, St. Louis, MO). Briefly, thiacremonone (1 mg) was dissolved in 1 ml of coupling buffer (0.1 M NaHCO3 and 0.5 M NaCl, pH 6.0). The CNBr-activated Sepharose 4B was swelled and washed in 1 mM HCl through a sintered glass filter, then washed with the coupling buffer. CNBr-activated Sepharose 4B beads were added to the thiacremonone-containing coupling buffer and incubated at 4°C for 24 hr. The thiacremonone-conjugated Sepharose 4B was washed with three cycles of alternating pH wash buffers (buffer 1, 0.1 M acetate and 0.5 M NaCl, pH 4.0; buffer 2, 0.1 M Tris-HCl and 0.5 M NaCl, pH 8.0). Thiacremonone-conjugated beads were then equilibrated with a binding buffer (0.05 M Tris-HCl and 0.15 M NaCl, pH 7.5). The control unconjugated CNBr-activated Sepharose 4B beads were prepared as described above in the absence of thiacremonone. The cell lysate or PRDX6 recombinant protein (Abnova, Taipei, Taiwan) were mixed with thiacremonone-conjugated Sepharose 4B or Sepharose 4B at 4°C for 24 hr. The beads were then washed three times with TBST. The bound proteins were eluted with SDS loading buffer. The proteins were then resolved by SDS-PAGE followed by immunoblotting with antibodies against PRDX6 (1∶1,000 dilution, Abcam, plc. Cambridge, UK).

### Ethics Statement

All experiments were approved and carried out according to the Guide for the Care and Use of Animals [Animal Care Committee of Chungbuk National University, Korea (CBNUA-436-12-02)].

### Animal Experiment

12-week-old C57BL/6J-Tg(Prdx6) mice were purchased from Jackson Lab (Maine, USA) and 12-week-old C57BL/6 mice were purchased from Koatech (Pyeongtaek, Korea). The mice were divided into four groups. Lewis Lung Carcinoma (LLC) cells were injected s.c. (1.2×10^6^ tumor cells/0.1 mL PBS/animal) with a 27 gauge needle. After 10 days, two groups of mice (n = 10) were i.p. injected with thiacremonone (30 mg/kg in PBS and 0.01% DMSO) two times a week for 3 weeks. The control group of mice (n = 20) were treated with vehicle [PBS and 0.01% DMSO (i.p.)]) two times a week for 3 weeks. For subcutaneous tumors the maximum allowable size is 20 mm in diameter for a mouse, thus we sacrificed all mice before reaching maximum size Cervical dislocation was performed for euthanasia.

### Immunohistochemistry

All tissue specimens were fixed in formalin and paraffin-enclosed for examination. Sections 4 μm thick were stained with hematoxylin and eosin (H&E) and immunohistochemistry. The sections were then blotted and incubated with mouse monoclonal antibodies and were washed three times for 5 min each in PBS, and then incubated with secondary antibodies for 2 hr. After the slides were washed and developed with DAB, the slides were counterstained with hematoxylin, mounted in aqua-mount, and evaluated on a light microscope (Olympus, Tokyo, Japan).

### Data Analysis

The data were analyzed using the GraphPad Prism 4 ver. 4.03 software (GraphPad Software, La Jolla, CA). Data are presented as mean ± SD. The differences in all data were assessed by one-way analysis of variance (ANOVA). When the P value in the ANOVA test indicated statistical significance, the differences were assessed by the Dunnett's test. A value of P<0.05 was considered to be statistically significant.

## Results

### Binding between thiacremonone and PRDX6

The interaction was assessed in a pull-down assay using thiacremonone-Sepharose 4B beads, and after PRDX6 was detected by immunoblotting with anti-PRDX6. The results indicated that thiacremonone bound with recombinant PRDX6 protein or cell lysates containing PRDX6 protein from human NCI-H460 lung cancer cells. (Fig. 1B and C). To identity the binding site of thiacremonone to PRDX6, we performed a computational docking model of thiacremonone with PRDX6 under the Schrödinger docking program, Glide. The results from the docking studies suggested that thiacremonone might covalently bind with Cys 47 residue in PRDX6 catalytic site. The ribbon representation docking model of thiacremonone with PRDX6 indicated that the following interactions are possible in the catalytic site; the side chain of Thr 44, Met 127 and Val 46 of PRDX6 (Fig. 1E). We also found decreased binding of thiacremonone with mutant PRDX6 C47S compared to PRDX6 (Fig. 1D). As shown in Fig. 1F, molecular surface representation of a docking model where the binding pocket for thiacremonone is formed in PRDX6.

### Effect of Thiacremonone on PRDX6-Luciferase Activity and Expression and Activity of PRDX6

**Figure 2:**
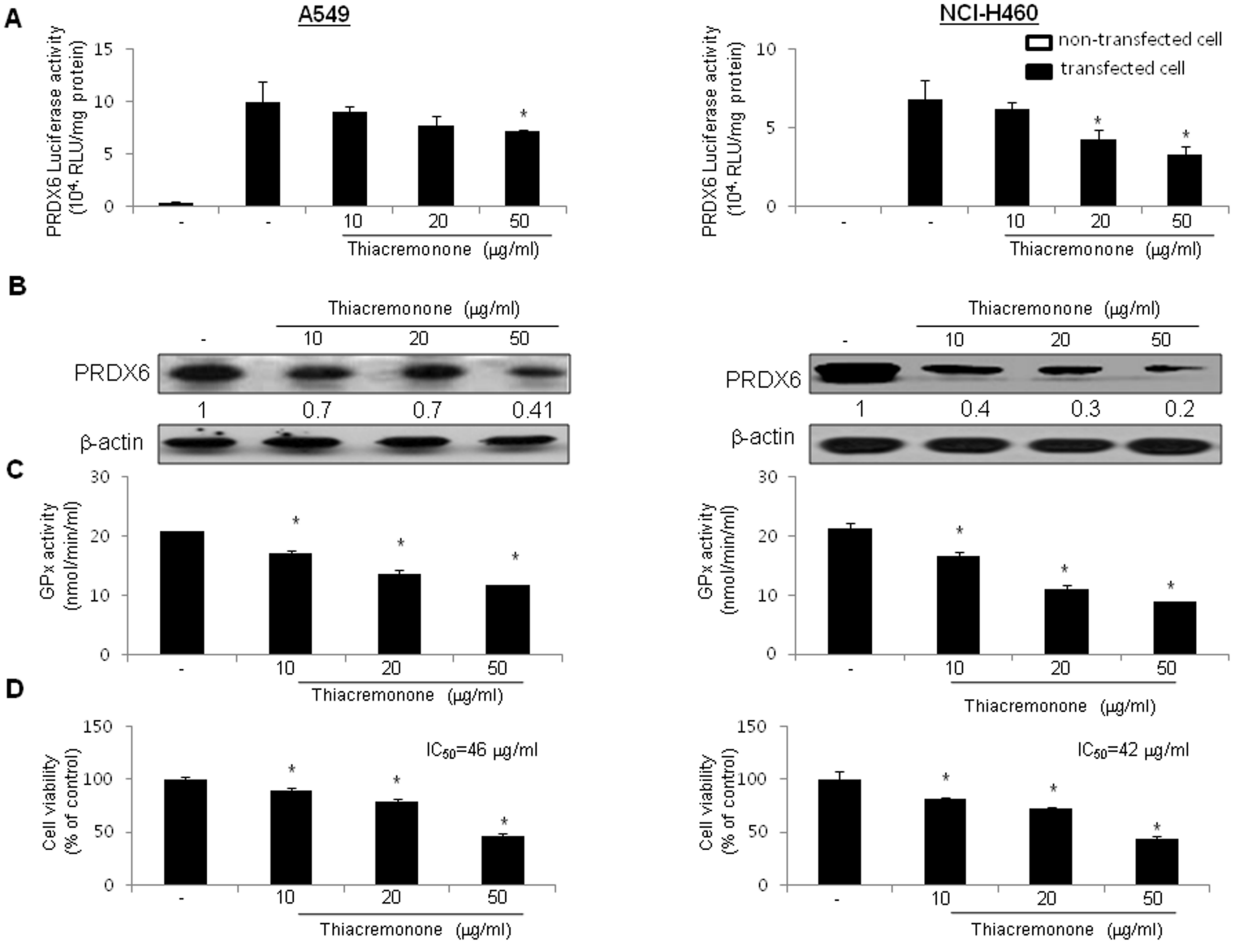
Effect of thiacremonone on prdx6-luciferase activity and expression and activity of PRDX6 and cell growth. (A) Lung cancer cells were transfected with prdx6-luciferase plasmid, and then treated with thiacremonone for 6 hrs. Luciferase activity was then determined as described in Materials and Methods. (B) Lung cancer cells were treated with thiacremonone for 72 hrs. Cell extracts were analyzed by western blotting. Each image and band is representative of three independent experiments. (C) The levels of glutathione peroxidase activity in lung cancer cells were measured using assay kits, as described in Materials and methods. (D) The cells were harvested by trypsinization and stained with 0.2% trypan blue. Values (A, C and D) are mean ±S.D. * p<0.05 compared with significantly different from untreated control cells.

To test whether interaction between thiacremonone and PRDX6 could attenuate prdx6-mediated promoter activity and expression, the cells were treated with thiacremonone (10, 20, and 50 μg/ml) for 6 hrs in A549 and NCI-H460 human lung cancer cells transfected with a prdx6-dependent luciferase reporter construct. The luciferase activity of cancer cells transfected with prdx6 promoter-Luc plasmid was over 5*10^4^ RLU/mg proteins, and the treatment with thiacremonone caused the suppression of luciferase activity in cancer cells (Fig. 2A). Consistent with the inhibitory effect on luciferase activity, the expression of PRDX6 was also decreased by thiacremonone in cancer cells (Fig. 2B). We further confirmed the relative glutathione peroxidase activity by thiacremonone. Thiacremonone inhibited glutathione peroxidase activity of both lung cancer cells, 72 hrs after treatment with thiacremonone as shown in Fig. 2C. These results suggest that the binding of thiacremonone to PRDX6 leads to the inhibition of PRDX6 expression and activity.

### Effect of Thiacremonone on Cell Growth in a Variety of Cancer Cells Including Lung Cancer Cells

Since PRDX6 is implicated in lung cancer cell growth, we investigated whether interaction of thiacremonone and PRDX6 could inhibit PRDX6 activity, thereby inhibit cancer cell growth. To assess the inhibitory effect of thiacremonone on cell growth of lung cancer cells, A549 and NCI-H460, we analyzed cell viability by direct cell counting. The cells were treated with several concentrations of thiacremonone (10, 20, 50 μg/ml) for 72 hr. As shown in figure 2D, thiacremonone inhibited cell proliferation of lung cancer cells in a concentration-dependent manner. Seventy-two hour treatment of thiacremonone inhibited A549 cell growth with IC_50_ value of 46 μg/ml, and NCI-H460 cells growth with IC_50_ values of 42 μg/ml, respectively. Morphologic observation showed that the cells were gradually reduced in size and changed into a small round single cell shape with the treatment of thiacremonone in A549 cells and NCI-H460 cells. We confirmed normal cell growth inhibition using CCD-18 colon and LL24 lung normal cells. However, thiacremonone showed no cytotoxic effect in the normal cells (Figure S1).

### Effect of Thiacremonone on Apoptotic Cell Death and the Expression of Apoptotic Regulatory Proteins

**Figure 3:**
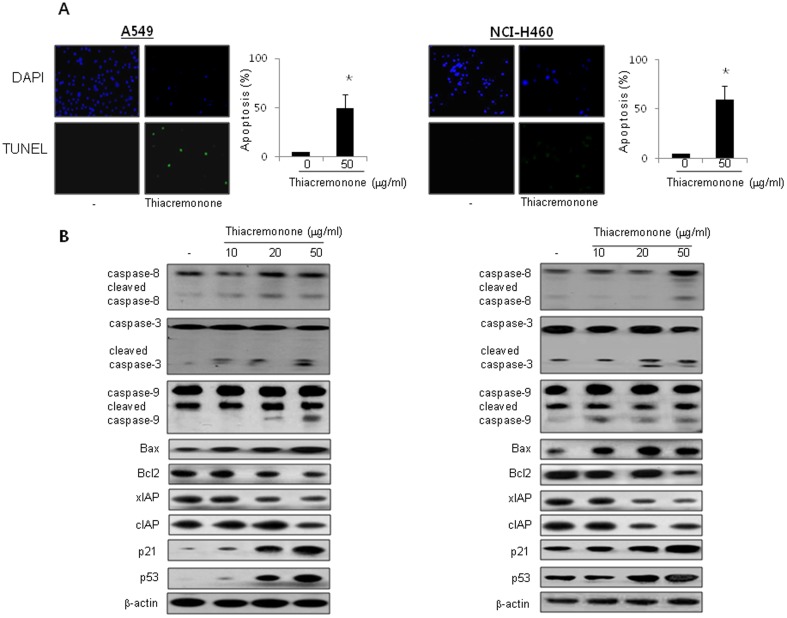
Effect of thiacremonone on apoptotic cell death and the expression of apoptosis regulatory proteins. (A) The lung cancer cells were treated in the absence (*left panels*) and presence of thiacremonone (50 μg/ml, *right pannels*) for 72 hrs, and then labeled with DAPI and TUNEL solution. Total number of cells in a given area was determined by using DAPI nuclear staining (fluorescent microscope). The green color in the fixed cells marks TUNEL-labeled cells. For quantification, three randomly selected areas were assessed. The apoptotic index (%) was determined as the (TUNEL-positive cell number/total DAPI stained cell number) x 100 (magnification, 200×). Values are mean ±S.D. * P<0.05 compared with significantly different from untreated control cells. (B) The lung cancer cells were treated with different concentrations of thiacremonone (10, 20, and 50 μg/ml) for 72 hrs. Expression of apoptosis regulatory proteins was determined using Western blot analysis. Each image and band is representative of three independent experiments.

Apoptosis is the process of programmed cell death which has an important role in anti-cancer effects of chemotherapeutics [18]. To determine that the inhibition of cell growth by thiacremonone was due to the induction of apoptotic cell death, we evaluated the changes in the chromatin morphology of cells by using DAPI staining followed by TUNEL staining assays. The double labeled cells were then analyzed by fluorescence microscope. Conversely with cell growth inhibition, DAPI-stained TUNEL-positive cells were significantly increased in thiacremonone treated cells. The treatment of thiacremonone resulted in about 45% and 65% induction of apoptotic cell death in A549 and NCI-H460 cancer cells, respectively (Fig. 3A). The activation of cell death regulatory proteins including caspases-9 and-3, as well as Bax, leads to apoptosis in cancer cells [19]. To figure out the expression of cell death regulatory proteins by thiacremonone, the expression of apoptotic cell death related proteins was investigated by Western blots. The expression of pro-apoptotic proteins, Bax and cleaved form of caspase-3, -8, -9, and p21 and p53 were increased by a treatment of thiacremonone. However, the expression of Bcl2, xIAP, and cIAP2 were decreased by a treatment of thiacremonone (Fig. 3B).

### Reverse of Inhibitory Effect of Thiacremonone on Cancer Cell Growth by Transfection of Mutant PRDX6 (C47S)

**Figure 4:**
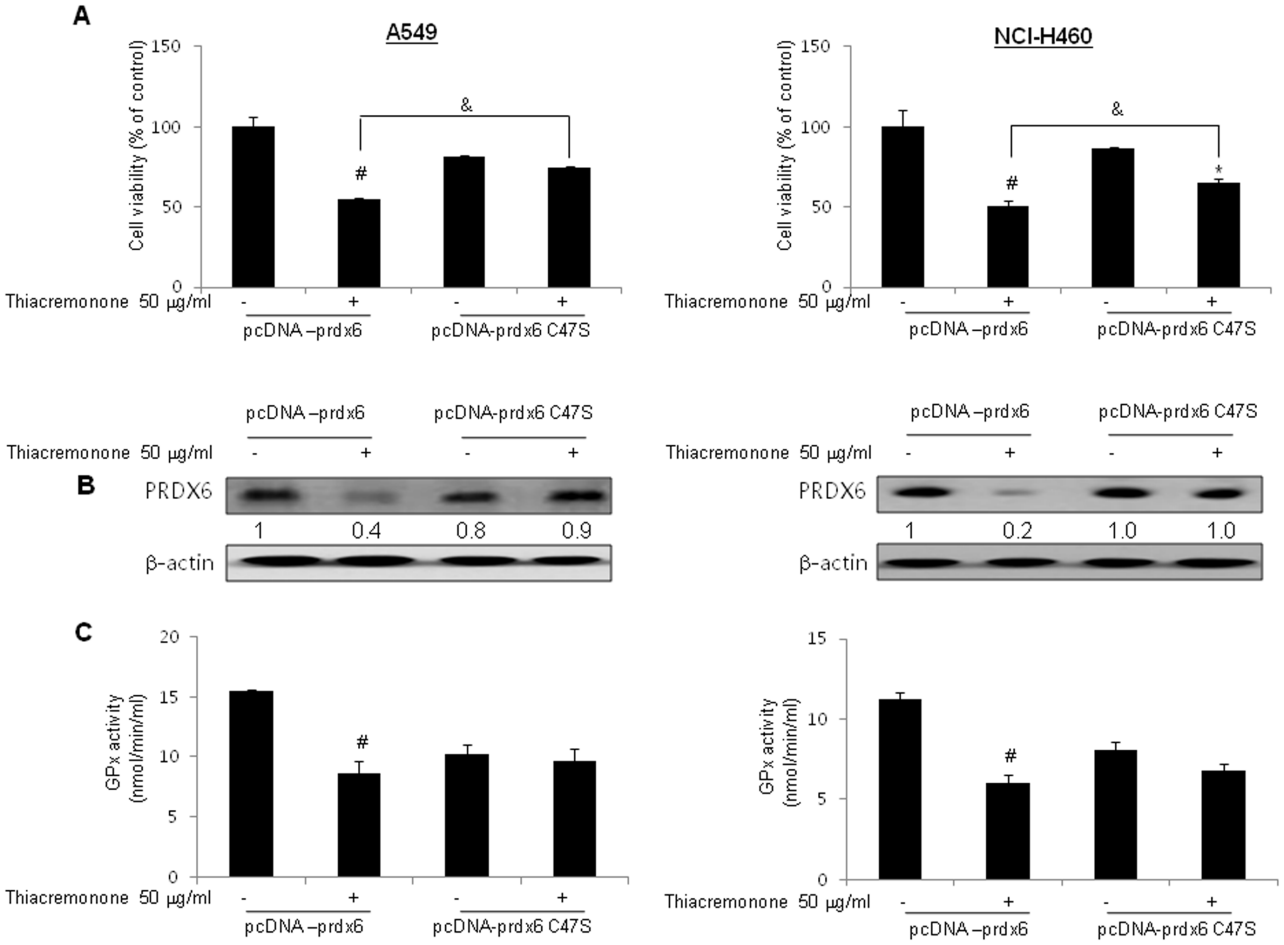
Reverse of inhibitory effect of thiacremonone by transfection of mutant prdx6 (C47S). (A) Lung cancer cells were transfected with pcDNA-prdx6 or pcDNA-prdx6 C47S, and then, thiacremonone was treated (50 μg/ml) for another 72 hrs. The cells were harvested by trypsinization and stained with 0.2% trypan blue. (B) Cell extracts were analyzed by western blotting. Each image and band is representative of three independent experiments. (C) The levels of glutathione peroxidase activity in lung cancer cells were measured using assay kits, as described in Materials and methods. Values are mean ±S.D. #, p<0.05 significantly different from untreated control cells. *, p<0.05, significantly different from untreated control cells transfected with pcDNA-prdx6 C47S. ^&^, p<0.05, significantly different between pcDNA-prdx6 and pcDNA-prdx6 C47S treated with thiacremonone.

**Figure 5:**
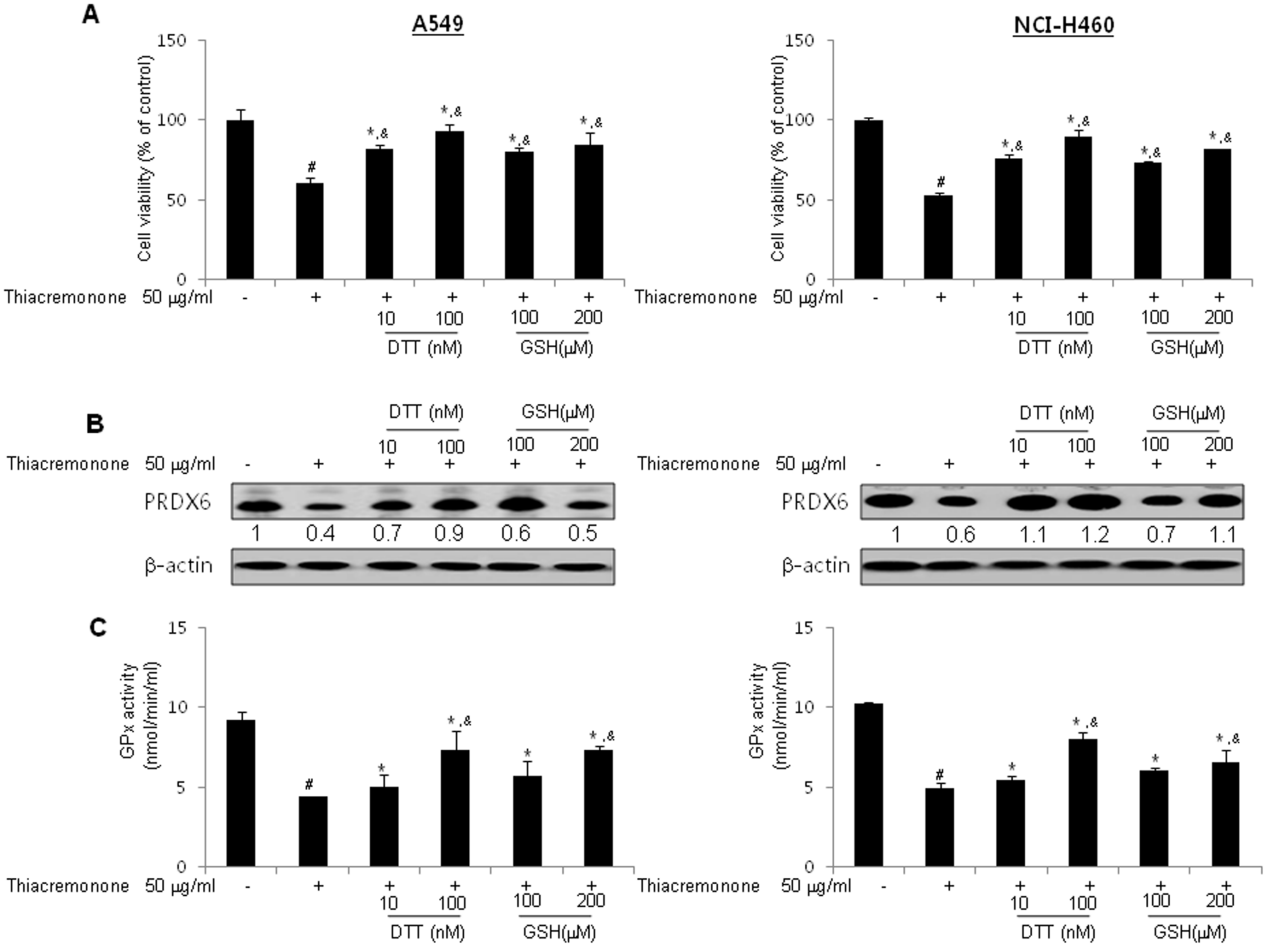
Reverse of inhibitory effect of thiacremonone by DTT and glutathione GSH. (A) Lung cancer cells were co-treated with indicated concentrations of DTT (10 and 100 nM) or GSH (100 and 200 μM) with thiacremonone (50 μg/ml) for 72 hrs. The cells were harvested by trypsinization and stained with 0.2% trypan blue. (B) Cell extracts were analyzed by western blotting. Each image and band is representative of three independent experiments. (C) The levels of glutathione peroxidase activity in lung cancer cells were measured using assay kits, as described in Materials and methods. Values (A and C) are mean ±S.D. #, p<0.05 compared with significantly different from untreated cells with thiacremonone. *, p<0.05, significantly different from untreated cells with DTT or GSH.. ^&^, p<0.05, significantly different between treated cell with thiacremonone and co-treated cells with DTT or GSH and thiacremonone.

To investigate whether cell growth was inhibited by the interaction of thiacremonone and PRDX6, the lung cancer cells were transfected with C47S-prdx6. The inhibitory effect of thiacremonone on cancer cell growth is reversed by mutant PRDX6 (C47S), and expression and activity of PRDX6 is also reversed (Fig. 4). These results suggest that the interaction of PRDX6 with thiacremonone could be significant for lung cancer cell growth, and thiacremonone suppresses lung cancer cell growth (Fig. 4A) via suppression of PRDX6 expression (Fig. 4B) and glutathione peroxidase activity (Fig. 4C). Furthermore, DTT and GSH suppressed the inhibitory effects of thiacremonone on cell growth, PRDX6 expression and glutathione peroxidase activity (Fig. 5). These data demonstrated that thiacremonone inhibited cell growth via inhibition of glutathione peroxidase activity and expression of PRDX6.

### Thiacremonone Inhibited Tumor Growth in Vivo Allograft

**Figure 6:**
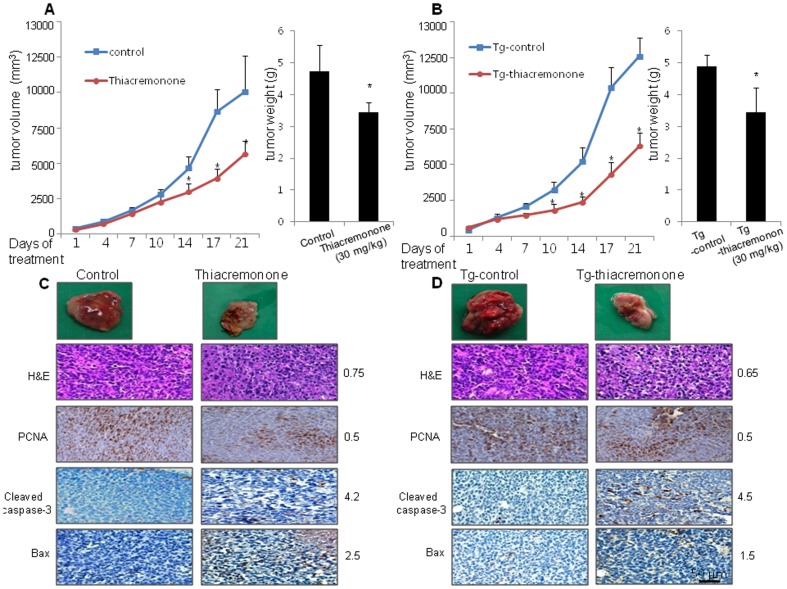
Effect of thiacremonone on tumor growth in allograft model. (A) Tumor volumes, weights, and images of normal mice. (B) Tumor volumes, weights, and images of PRDX6 overexpressed mice. Values (A and B) are mean ±S.D. * P<0.05 significantly different from untreated mice. (C) Tumor sections of normal mice were analyzed by H&E stain and expression of proteins by immunohistochemistry. The resultant tissues were developed with DAB, and counterstained with hematoxylin. (D) Tumor sections of PRDX6 overexpressed mice were analyzed by H&E stain and expression of proteins by immunohistochemistry. The resultant tissues were developed with DAB, and counterstained with hematoxylin. For quantification, 200 cells at three randomly selected areas were assessed, and the specific protein positively stained cells were counted. Scale bar indicates 50 μm.

To elucidate the anti-tumor effect of thiacremonone in vivo, the tumor growth in allograft bearing mice following thiacremonone treatments was investigated. In LLC allograft studies, thiacremonone was administrated intraperitoneally twice per week for 3 weeks to mice. Tumor volume was measured weekly, and all mice were killed at the end of experiment when tumors were dissected and weighted. The inhibitory effect of thiacremonone on the growth of lung tumor was significant in both allograft models using C57BL/6J mice as well as PRDX6 overexpressed mice. The relative tumor growth was measured after treatment of thiacremonone (30 mg/kg, n = 10). Tumor growth in C57BL/6J (tumor volume; 8000.4±2000.7 mm^3^, tumor weigh; 4.7±0.82 g) mice was significantly decreased by thiacremonone (tumor volume; 4543.6±731.1 mm^3^, tumor weight; 3.45±0.31 g, Fig. 6A). This inhibitory effect was found in PRDX6 overexpressed mice (tumor volume; 10060.5±1039.6 mm^3^ (Tg-control) versus 5048.9±737.9 mm^3^ (Tg-thiacremonone), tumor weight; 4.9±0.21 g (Tg-control) versus 3.44±0.78 g (Tg-thiacremonone), Fig. 6B). The immunohistochemistry analysis of tumor section by H&E, and by proliferation antigens against PCNA staining revealed that thiacremonone inhibited tumor growth, but assessments of pro-apoptotic proteins, Bax and cleaved caspase-3 by IHC revealed more frequently in thiacremonone-treated LLC bearing C57BL/6J mice and PRDX6 overexpressed mice (Fig. 6C and D).

### Effect of Thiacremonone on Expression and Activity of PRDX6, and Cell Death Regulatory Proteins

To figure out the expression of PRDX6 and apoptotic cell death regulatory proteins by thiacremonone, expression of proteins were investigated by Western blots. PRDX6 was decreased by treatment of thiacremonone in both normal and PRDX6 overexpressed mice tumor tissue. The expression of pro-apoptotic proteins, Bax and cleaved form of caspase-3, -8, -9, as well as p21 and p53 was increased, whereas the expressions of Bcl2, xIAP, and cIAP2 were decreased by treatment of thiacremonone in both normal and PRDX6 overexpressed mice tumor tissues (Figure S2A). As shown in Figure S2B, thiacremonone also inhibited glutathione peroxidase activity of PRDX6 in normal and both PRDX6 overexpressed mice tumor tissues.

## Discussion

Many studies have shown that fresh garlic extracts, aged garlic, garlic oil and specific organosulfur compounds generated by processing garlic could alter carcinogen metabolism, inhibit tumor cell growth through induction of cell cycle arrest, apoptosis, and prevention of promotion and angiogenesis [20]–[21] in a variety of cancer cell lines including hepatoma, cervical, prostate, lung, colon cancer cells [22]–[26]. Diallyl trisulfide (DATS), a sulfane sulfur-containing compound showed the strongest inhibition of cell proliferation and the greatest induction of caspase-3 activity in HepG2 cells [23]. DATS suppresses human lung cancer cell growth by causing G2-M phase cell cycle arrest followed by Bax-mediated apoptosis [26]. Diallyl sulfide (DAS) induces cell cycle arrest and apoptosis through the p53, caspase- and mitochondria-dependent pathways in HeLa human cervical cancer cells [25]. Allicin (diallyl thiosulfinate) induces apoptosis in colon cancer cells [22]. Thiacremonone was isolated as a sulfurcompound from a hot water extract of garlic, and found that this compound could have an anti-cancer effect on colon cancer [27]. Our present data showed that a seventy-two hour treatment of thiacremonone induced cell death of lung cancer cells with an IC_50_ value of 46 μg/ml in A549 cells, and 42 μg/ml in NCI-H460 cells, respectively. Lung cancer cell growth was significantly decreased by DATS in a concentration- and time-dependent manner with an IC_50_ of <3.6 μg/ml [26], and with an IC_50_ of 7.3 μg/ml by DADS [34]. The IC_50_ of S-allylcysteine (SAC) to human metastatic cells was about 5.3 mg/ml at day 3 [28]. After treatment for 24 hr, the cell growth of prostate cancer cells with 9 μg/ml sulforaphane (SFN) was 10.2±3.1% compared with that in control cells (100%) [29]. Even though concentrations of compounds leading cancer cell growth inhibition are different, it depends on the cell type and compounds treated, derived compounds from garlic including thiacremonone have an anti-cancer effect. However, exact action mechanisms are still unclear.

As PRDX6 scavenge peroxide, such as small H_2_O_2_, it supports survival of cancer cells and tumor maintenance [30]. Many studies have demonstrated that PRDX6 promotes invasion and metastasis of a variety of cancer cells including lung, breast, and ovarian cancer cells [4], [12], [31]. PRDX6 is expressed in all major organs, with a particularly high level in the lung [32]. Moreover, PRDX6 was found at higher levels in lung squamous cell carcinoma patients [33]. Overexpression of PRDX6 increased lung cancer cell growth via activity of PRDX6 [4]. Thus, targeting PRDX6 by certain compounds could be effective for lung tumor growth inhibition as chemotherapeutics. PRDX6 has dual enzyme activities such as glutathione peroxidase and iPLA2 [32]. Glutathione peroxidase promotes cancer cell growth via inhibition of apoptosis and a higher probability or of relapses including lung metastasis and local recurrences [34]. Glutathione peroxidase inhibits cisplatin-induced apoptosis via the down-regulation of Bcl2 in NCI-H460 human lung cancer cells [35]. In the tumor tissues, glutathione peroxidase activity was higher than in the tumor-free tissues [36]. Our data showed that decreasing glutathione peroxidase activity in lung cancer cells was associated with lung cancer cell growth inhibition in concentration-dependent manner. Expression of PRDX6 was decreased by thiacremonone in both in vitro and in vivo. In addition, by the pull-down assay using thiacremonone-agarose bead, we found that thiacremonone bound with recombinant PRDX6 protein or cell lysates containing PRDX6 from human NCI-H460 lung cancer cells, but the binding was not observed in the cell lysates extracted from the cancer cells transfected with C47S-prdx6 mutant plasmid. The cell growth inhibitory effect of thiacremonone was also significantly abolished in cells transfected C47S-prdx6 mutant plasmid. These results indicate that thiacremonone inhibits lung cancer cell growth through inhibition of glutathione peroxidase of PRDX6 by interaction of Cys-47 of PRDX6.

In in vivo study with LLC allograft bearing mice, treatment of thiacremonone (30 mg/kg injected intraperitoneally twice a week for 3 weeks) significantly inhibited tumor growth by approximately 50–60%. The immunohistochemistry analysis of tumor section by H&E, and by proliferation antigens against PCNA staining revealed that thiacremonone inhibited tumor growth. In addition, our data also showed that thiacremonone inhibited expression of PRDX6 accompanied with inhibition of glutathione peroxidase activity in lung tumor tissues. Moreover, expression of pro-apoptotic proteins, cleaved form of caspase-3 and Bax, was increased and anti-apoptotic protein, but expression of Bcl2, cIAP, and xIAP was decreased by treatment of thiacremonone. Our results showed that thiacremonone showed no cytotoxic effect in the normal CCD-18 Co colon, and LL24 lung normal cells. These data suggest that thiacremonone may be potentially beneficial for anti-cancer effect with comparatively low toxicities via interaction with PRDX6.

## Supporting Information

Figure S1 **No cytotoxic of thiacremonone in normal cells.** After treatment of thiacremonone for 72 hrs, the cells were harvested by trypsinization and stained with 0.2% trypan blue. Relative cell survival rate was determined by counting live and dead cells. The results were expressed as a percentage of viable cells. Values are mean ±s.d. * p<0.05 compared with significantly different from untreated control cells.
(TIF)

Figure S2 **Effect of thiacremonone on the expression of apoptosis regulatory proteins and activity of PRDX6 in tumor tissue.** (A) Tumor extracts from three mice were analyzed by western blotting. Each image and band is representative of three independent experiments. (B) The levels of glutathione peroxidase activity in tumor tissue were measured using assay kits, as described in Materials and methods. Values are mean ±s.d. * P<0.05 significantly different from untreated mice.
(TIF)
